# Interdisciplinary Approaches towards Materials with Enhanced Properties for Electrical Engineering

**DOI:** 10.3390/polym8080307

**Published:** 2016-08-16

**Authors:** Frank Wiesbrock

**Affiliations:** Polymer Competence Center Leoben, Roseggerstrasse 12, Leoben 8700, Austria; frank.wiesbrock@pccl.at; Tel.: +43-3842-42962-42

**Keywords:** epoxy resin, nanocomposites, high-voltage engineering, mica/epoxy composites, crosslinked polyethylene (XLPE), mechanical properties, charge transport, surface chemistry

The *internationally growing demand for electrical energy* is one of the most prominent triggers stimulating research these days. In this *highly interdisciplinary research area*, electrical engineers, material scientists and chemists collaborate for the design and fabrication of the next generation of high-voltage machinery and electro-technical devices. *Nanocomposites with enhanced thermal conductivity and improved electric properties* are in the center of these joint research activities. Hence, in this Special Issue of the open-access journal *Polymers*, the state-of-the-art research and technology of the area ‘*micro- and nanocomposites for electrical engineering applications*’ has been summarized in three review articles, while the current research trends and the development and characterization of novel materials have been described in eight original research articles.

**State-of-the-art of research and technology.** The comprehensive review article by Plesa et al. addresses with dedication the *structure-property relationships of composite materials with special respect to their electric properties* and the resulting potential application fields [1]. Originating from the high demands regarding the reliability and lifetime expectance of high-voltage engineering machineries such as generators and transformers, *the mechanical properties of insulating resins and the corresponding micro- and nanocomposites* are of prime importance; this topic has been summarized in the review article by Moser and Feuchter [2], in which epoxy-based resins are discussed in detail. *Mica/epoxy composites* are the most commonly used insulation materials in *high-voltage rotating machines*, and their properties as well as the *possibly occurring failure mechanisms* of the composite material have been described in the review article by Andraschek and colleagues [3].

**Novel composites based on epoxy resins or on low-density polyethylene.** The preparation as well as the isotropic electrical properties (in the case of additionally present silver nanowires) of *epoxy-based nanocomposites containing in-situ formed silver nanoparticles* was reported by Kang, Lee, and Song [4]. Vaughan and Yeung described the effect of *siloxane-mediated surface functionalization of silica nanoparticles* [5]; the corresponding epoxy-based nanocomposites exhibited breakdown strengths that were increased by approx. 50% compared to the unfilled epoxy resin. Gubanski et al. reported the *decrease of the direct current conductivity of low-density polyethylene composites* containing inorganic fillers such as magnesium oxide and aluminum oxide [6]. In a subsequent study [7], a *bipolar charge transport model* was employed to investigate this reduction, from which the *reduced charge injection at electrodes* was identified as the most important parameter causing the observed effects.

**Materials based on polymers from renewable resources and composites based on (per-) fluorinated polymers.** Smit, Wiesbrock et al. reported the synthesis of crosslinkable copoly(2-oxazoline)s from *fatty acids such as castor oil and coconut oil* [8]. The crosslinked copolymers exhibited *electric properties similar to those of polyamides*, which renders them medium insulators. The in-situ preparation of *reduced graphene oxide/carboxymethyl chitosan composites* was described by Luo, Chen, and colleagues [9]. An electrode modified with this composite showed a *high detection performance for bivalent copper ions*. Nanocomposites of *poly (vinylidene fluoride) and cube-shaped surface-hydroxylated Ba_0.6_Sr_0.4_TiO_3_ nanoparticles* were reported by Zhai et al. to show an *increased dielectric constant and improved breakdown strength* compared to the unfilled polymer [10]. Mariotto and colleagues described composites of *Nafion and nano-sized sulfated titanium dioxide* [11], in which they found that the inclusion of 2 wt % of fillers yielded structures that consisted of *filler-rich regions, which were separated by areas of almost pure Nafion*. This structural arrangement does not easily provide any *proton percolation path*, and, hence, a higher resistance was expected for this composite.

In summary, this Special Issue of *Polymers* compiles the current state-of-the-art of research and technology in the area of ‘*micro- and nanocomposites for electrical engineering applications*’ and highlights prominent current research directions in the field. We very much hope that you enjoy reading it.
